# Electrochemical biosensors for saxitoxin detection: advances, limitations, and challenges for market transition

**DOI:** 10.1007/s10661-026-15797-x

**Published:** 2026-08-18

**Authors:** Isadora Bernardes Sequalini, Thiago Teixeira da Silva, Larissa Victoria do Carmo de Oliveira, Nirton Cristi Silva Vieira

**Affiliations:** Institute of Science and Technology, Federal University of São Paulo, São José Dos Campos, SP 12231-280 Brazil

**Keywords:** Saxitoxin, Electrochemical biosensors, Detection, Contamination

## Abstract

Saxitoxins, produced by freshwater cyanobacteria and marine dinoflagellates, block neuronal sodium channels and can cause paralysis or death. They contaminate drinking water, bioaccumulate in aquatic organisms, and pose serious health risks. Conventional detection methods, though reliable, are costly, time-consuming, and require specialized personnel, limiting rapid or on-site use. Biosensors have emerged as attractive alternatives, with electrochemical platforms standing out for their high sensitivity, low cost, miniaturization potential, and easy integration into portable devices. These advantages make them promising tools for in situ monitoring of saxitoxins and justify a dedicated review. This work critically analyzes electrochemical biosensors for STX detection, highlighting advances, limitations, and challenges toward commercialization. We discuss operating principles, recognition elements, analytical performance, and classify devices by electrochemical technique (potentiometric, impedimetric, voltammetric), providing a comparative perspective on their respective strengths and limitations. Despite remarkable sensitivity—often below regulatory limits—key obstacles remain: lack of fabrication standardization, poor long-term stability, limited validation with naturally contaminated samples, insufficient robustness for complex matrices, low reproducibility, and absence of regulatory approval pathways. In addition, challenges associated with fabrication scalability and interlaboratory reproducibility continue to hinder the transition from proof-of-concept devices to commercially viable technologies. Overcoming these barriers is crucial for translating laboratory prototypes into reliable, field-deployable devices for environmental monitoring and food safety applications. Future developments should focus on improving robustness, reproducibility, and analytical validation to accelerate the practical implementation of electrochemical STX biosensors.

## Introduction

Paralytic shellfish toxins (PSTs) comprise a family of approximately 60 structurally related neurotoxins, including saxitoxin (STX), neosaxitoxin (neoSTX), gonyautoxins (GTXs), and decarbamoylsaxitoxin (dcSTX), that vary in potency but share the ability to block neuronal sodium channels, potentially causing paralysis and death (Ballot et al., 2016). Produced by filamentous cyanobacteria in freshwater and by marine dinoflagellates, PSTs contaminate drinking water and accumulate in aquatic organisms, threatening both human and animal health. International guidelines reflect their toxicity, with the World Health Organization (WHO) establishing a maximum concentration of 3.0 µg/L for STX in drinking water (Chorus & Welker, 2021), while food safety agencies setting limits of 80 µg STX equivalents per 100 g of shellfish tissue, based on the toxicity equivalency factors of different analogs, with STX itself representing the basic structure used as the reference compound (Arnich & Thébault, 2018). Accordingly, this review focuses on the electrochemical detection of STX, with analytical results generally expressed as STX equivalents, following current regulatory practice.

Given their high toxicity and strict regulatory limits, the ability to detect STX rapidly and accurately is essential to prevent intoxication outbreaks and ensure the safety of drinking water and food products. Conventional methods such as high-performance liquid chromatography (HPLC), liquid chromatography coupled with mass spectrometry (LC–MS), and immunoassays (e.g., ELISA), though reliable and widely used, are costly, time-consuming, and require specialized personnel and infrastructure (Kaushik & Balasubramanian, 2013). Much of this time demand arises from extensive sample-preparation steps, including cyanobacterial cell lysis, tissue homogenization, and multistep extraction procedures needed to release and isolate STX before analysis. These requirements hinder rapid or decentralized monitoring and highlight the need for portable, cost-effective detection strategies (Ishak et al., 2023).

Biosensors are widely regarded as a promising strategy for the detection of diverse analytes in a rapid, sensitive, and cost-effective way. This potential has been shown by several reviews that have reported significant advances for toxin detection, for example, microcystins (Dai et al., 2025; Pang et al., 2020; Vogiazi et al., 2019). A sensor is generally defined as a device that produces a measurable signal in response to a physical or chemical property (Eggins, 2007). A biosensor, however, integrates a biological or biomimetic recognition element with a transducer, allowing for the specific qualitative or quantitative analysis of a target analyte within a sample. Biosensors can therefore represent an effective solution for STX detection, particularly for rapid and cost-effective monitoring at the point of contamination (Eggins, 2007).

Some types of biosensors have been investigated for STX detection, including optical (Gao et al., 2017), piezoelectric (Bergantin & Sevilla III, 2010), colorimetric (Qiang et al., 2020), and electrochemical transducers (Park et al., 2022). Among these, electrochemical biosensors have emerged as the dominant approach in academic research and the most promising for commercial translation, owing to their combination of high sensitivity, cost-effectiveness, and compatibility with miniaturization and integration into portable devices with relatively simple electronics (Ronkainen et al., 2010). Also, unlike other biosensors, electrochemical systems enable the use of multiple complementary techniques (e.g., voltammetry, impedance, potentiometry) within the same device, facilitating optimization of analytical performance. Nevertheless, despite encouraging laboratory results, no electrochemical biosensor for STX detection has yet reached the market, highlighting the gap between academic prototypes and field-ready solutions.

Although progress has been made in the biosensing of cyanotoxins, the detection of STX remains particularly challenging due to intrinsic analytical limitations. Unlike many electroactive compounds, STX lacks significant redox activity, which hinders direct electrochemical detection and often requires signal amplification strategies or selective biorecognition elements (Kaushik & Balasubramanian, 2013; Li & Persson, 2021). STX belongs to a family of over 50 analogs of mollusk paralytic toxin with very similar structures and toxicological properties, further increasing the difficulty of achieving selective recognition (Raposo et al., 2020). Matrix effects from environmental water sources and seafood extracts further complicate the analysis and often negate sample pretreatment procedures, limiting direct field applications (Li & Persson, 2021). Consequently, challenges related to selectivity, robustness, and validation under real-world conditions still hinder the transition of STX biosensors from proof-of-concept devices to commercially viable technologies.

Despite the toxicity of STX and their significant impact on public health, electrochemical biosensors for STX detection have not been reviewed until now. In contrast, microcystin detection has been extensively studied, with several reviews recognizing biosensors as a promising alternative (Dai et al., 2025; Pang et al., 2020; Vogiazi et al., 2019). This discrepancy highlights that monitoring STX through electrochemical technologies remains an emerging and fragmented field that demands greater scientific attention. This review, therefore, addresses this gap by providing a comprehensive and critical synthesis of electrochemical biosensors for STX detection. Devices are categorized according to the type of electrochemical transduction employed (potentiometric, impedimetric, or voltammetric), and their analytical performance is systematically compared, considering parameters such as limit of detection, operating range, stability, and validation in real samples. In contrast to previous descriptive overviews, this article emphasizes cross-study comparisons, identifies recurring strengths and limitations, and highlights key research gaps that hinder translation from laboratory prototypes to field-ready devices. To facilitate this analysis, we also include summary tables and original schematic illustrations that condense the state of the art and outline future directions for the development of robust, practical biosensors for STX monitoring.

## Electrochemical biosensors for STX

### Potentiometric biosensors/sensors

Traditional potentiometric biosensors detect analytes by monitoring changes in the potential of an electrochemical cell driven by specific recognition events (e.g., antigen–antibody binding). The signal arises from variations in the working-electrode potential, where the biorecognition element is immobilized, and measurements are performed at open-circuit potential (OCP), i.e., with negligible current flow. Potentiometric biosensors provide precise and selective detection across a range of analytes, making them well-suited to real-time monitoring systems (Walker et al., 2021).

**Fig. 1:**
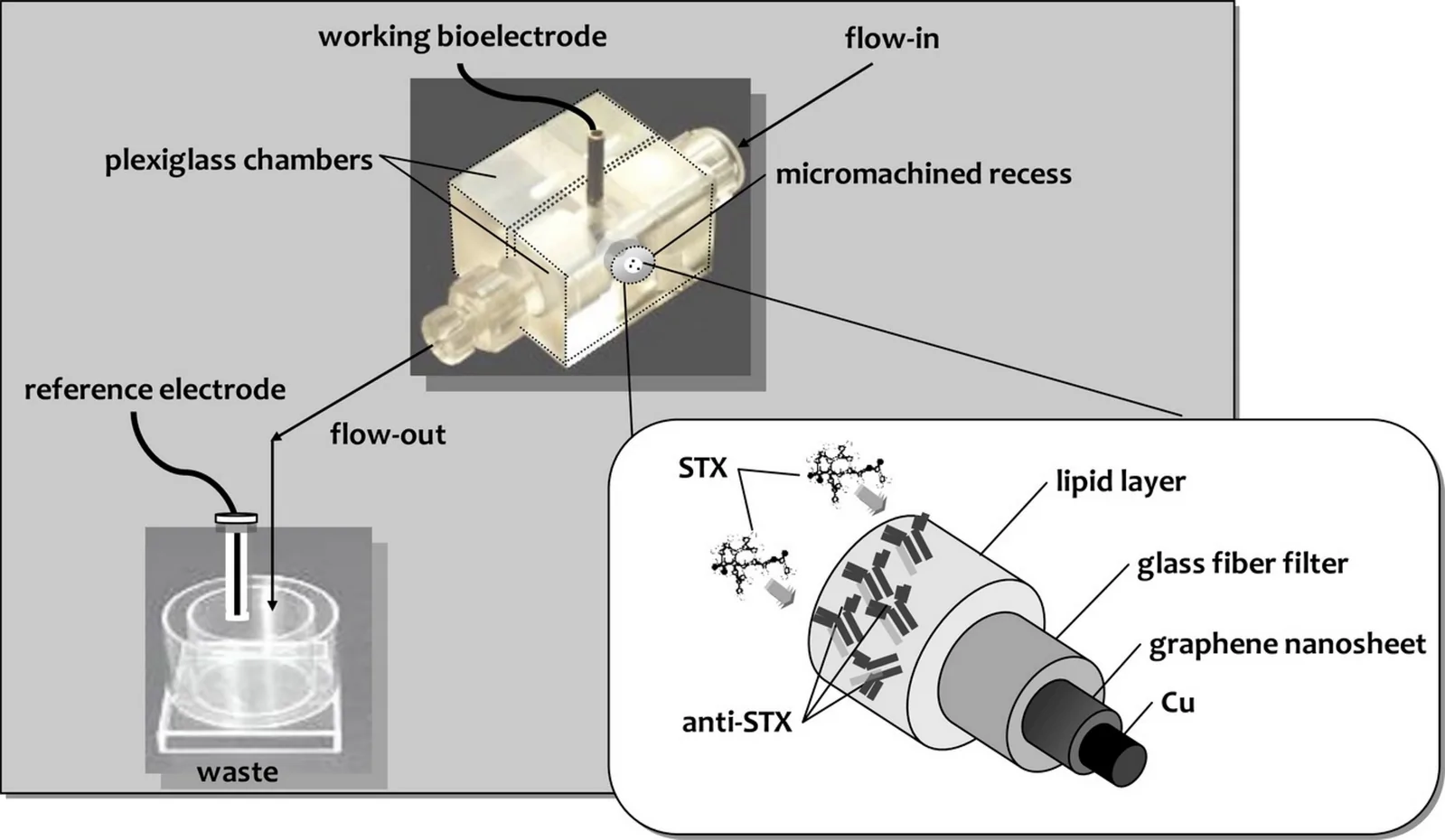
Schematic configuration of the saxitoxin electrochemical biosensor and the bioelectrode edge surface (Reprinted with permission from Bratakou et al., 2017. Copyright 2017 Wiley-VCH)

Bratakou et al. developed a potentiometric biosensor for STX detection that integrates graphene nanosheets with polymer-stabilized lipid films incorporating anti-STX antibodies (Bratakou et al., 2017). The biosensor showed good reproducibility, fast response, extended operational lifetime, and a Nernstian sensitivity of ~ 60 mV/dec over the tested concentration range. Additionally, the device was evaluated in spiked mussel and lake water samples. The flow-cell design facilitates STX capture during sample injection, where conformational changes in the lipid layer are transduced into potentiometric signals. A reference electrode completes the circuit. The biosensor demonstrated a limit of detection (LOD) of 1 nmol/L (~ 0.3 µg/L) with rapid response times (5–20 min). Validation tests were performed using STX-spiked freshwater from a natural reservoir and shellfish extracts (mussels, clams, oysters), achieving reliable recoveries of 90–106% for the low concentration and 95–102% for the high concentration (Bratakou et al., 2017) (Fig. 1).

**Fig. 2:**
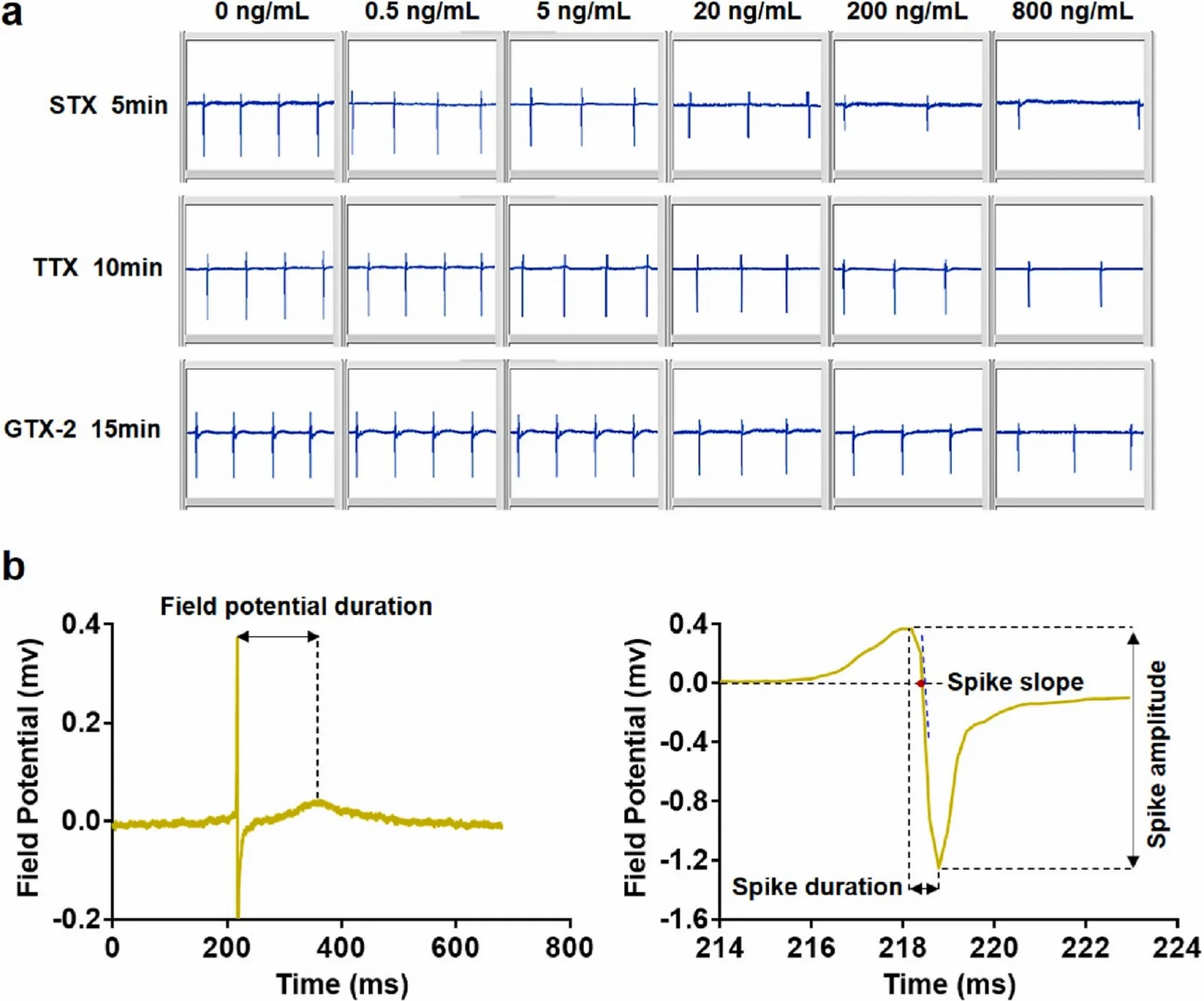
Concentration-dependent effects on cardiomyocyte-based biosensors after exposure to the sodium channel toxins at the optimal time points. **a** Representative changes of EFP waveforms after toxins treatment. **b** The typical EFP waveform and the feature parameters (reprinted with permission from Sun et al., 2023. Copyright 2023 Elsevier)

Other approaches have expanded the range of recognition elements beyond antibodies. For example, Sun et al. used cardiomyocytes as the recognition system, exploiting the interaction between STX and sodium channels (Sun et al., 2023). The main advantage of this biosensor lies in its ability to selectively detect different toxins such as STX, tetrodotoxin (TTX), and gonyautoxin-2 (GTX-2) based on their electrophysiological interactions (Bratakou et al., 2017). Figure 2 shows typical changes in extracellular field potentials (EFPs) of cardiomyocytes after exposure to STX, TTX, and GTX-2, highlighting key parameters such as spike duration and amplitude that enable differentiation among toxins. The device exhibited low LODs: 0.29 µg/L for STX, 0.30 µg/L for TTX, and 0.16 µg/L for GTX-2. To evaluate potential applications in real samples, the study employed synthetic systems by spiking purified toxins into toxin-free shellfish extracts (pre-screened by ELISA) (Sun et al., 2023).

The Rudnitskaya group developed potentiometric chemical sensors for STX detection based on the concept of an electronic tongue (e-tongue) (Ferreira et al., 2018). In the first study, miniaturized PVC-membrane sensors were engineered with calixarene and crown-ether ionophores tailored to interact with the guanidinium groups of STX. The sensors showed cross-sensitivity to STX, dcSTX, gonyautoxin-5 (GTX5), and C1&C2 (11α-hydroxy-sulfocarbamoyl-saxitoxin and 11β-hydroxy-sulfocarbamoyl-saxitoxin), while retaining high selectivity over common marine cations (Na^+^, K^+^, Ca^2+^). The LOD values ranged from 0.25 to 0.9 μmol/L (74.8 to 269.1 μg/L, considering STX molecular weight) for STX and dcSTX, and from 0.08 to 1.8 μmol/L for GTX5 and C1 and C2 (31.6 to 711.0 μg/L, considering GTX5 molecular weight), matching legal safety thresholds for bivalves (Ferreira et al., 2018).

Building on that platform, the group assembled six sensors into a potentiometric e-tongue and applied the partial least squares (PLS) regression for simultaneous quantification of the four toxins in mixed solutions. Individual sensors lacked sufficient selectivity, but the array enabled robust calibration and validation across toxins (Cruz et al., 2018). LODs for the array remained in the same ranges as in the first study, confirming suitability for concentrations around legal thresholds. A critical concern was addressing matrix effects from mussel extracts. To address this challenge, the team quantified the shift in the calibration curves between the buffer and mussel extracts. It implemented a calibration-transfer strategy (Joint-Y PLS) using five fortified samples to adapt the original PLS model to the new matrix. After transfer, e-tongue predictions matched dcSTX, GTX5, and C1 and 2 (no significant differences by *t*-test), confirming its practical viability for STX screening with the recalibration strategy (Cruz et al., 2018).

Electrolyte-insulator-semiconductor (ELIS) sensors constitute an alternative to conventional potentiometric sensors by detecting variations in surface potential at the electrolyte-insulator-semiconductor interface. The insulating layer (e.g., SiO_2_, S_3_N_4_, or Al_2_O_3_, etc.) governs charge distribution, enabling the transduction of interactions between the recognition system and the analyte into measurable electrical signals. ELIS operates as a label-free biosensor with high sensitivity and real-time monitoring capability, making it a powerful platform for selective analyte detection (Poghossian & Schöning, 2020).

**Fig. 3:**
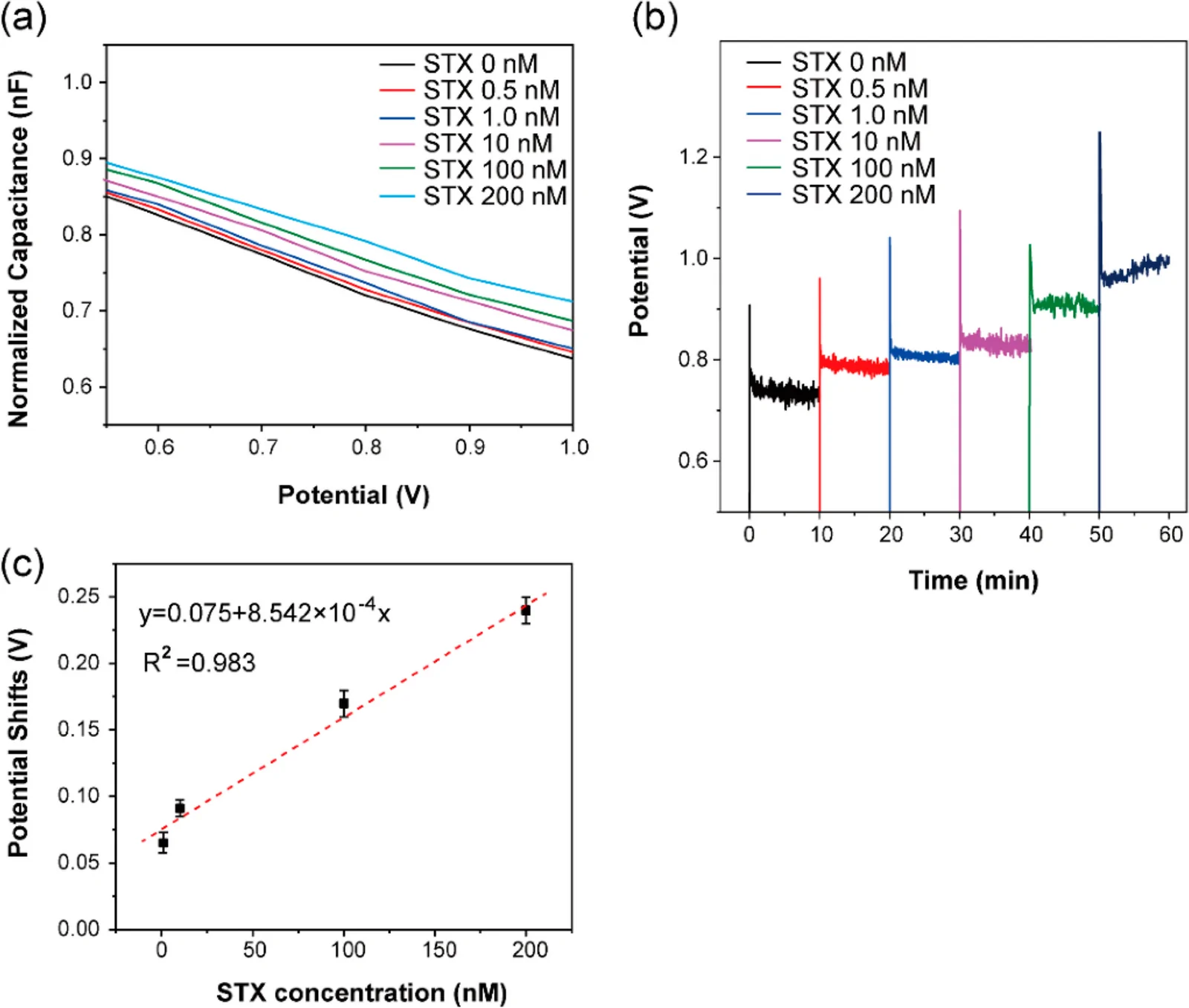
**a** *C-V* curves and **b** ConCap measurements of the biosensor to STX (from 1.0 to 200 nM). **c** Statistical results of ConCap measurements; the experiment was repeated three times (Reprinted from Ullah et al., 2021, under the terms of the Creative Commons Attribution License (CC BY 4.0). Copyright 2021 MPDI)

Ullah et al. reported the development of an ELIS biosensor based on the two-dimensional (2D) material Ti_3_C_2_T_x_ for the detection of STX (Ullah et al., 2021). Aptamers specific to STX were immobilized on the Ti_3_C_2_T_x_ surface, whose large surface area and abundant functional groups facilitated potent and selective interactions with both the aptamer and the target toxin. Sensor performance was evaluated using capacitance–voltage (C-V) curves (Fig. 3a) and constant capacitance (ConCap) mode (Fig. 3b), both of which revealed pronounced potential shifts with increasing STX concentrations over time (Fig. 3c). The device exhibited a linear detection range of 1.0–200 nmol/L with a LOD of 0.03 nmol/L (0.009 μg/L), well below regulatory thresholds. Its applicability to real samples was confirmed using ethanol-processed mussel tissue extracts spiked with 100 nM (30 μg/L) STX, achieving a recovery of 103%. In addition, selectivity against pectenotoxins and okadaic acid (OA), together with stability over 15 days, further underscores its potential for practical monitoring of STX (Ullah et al., 2021).

**Fig. 4:**
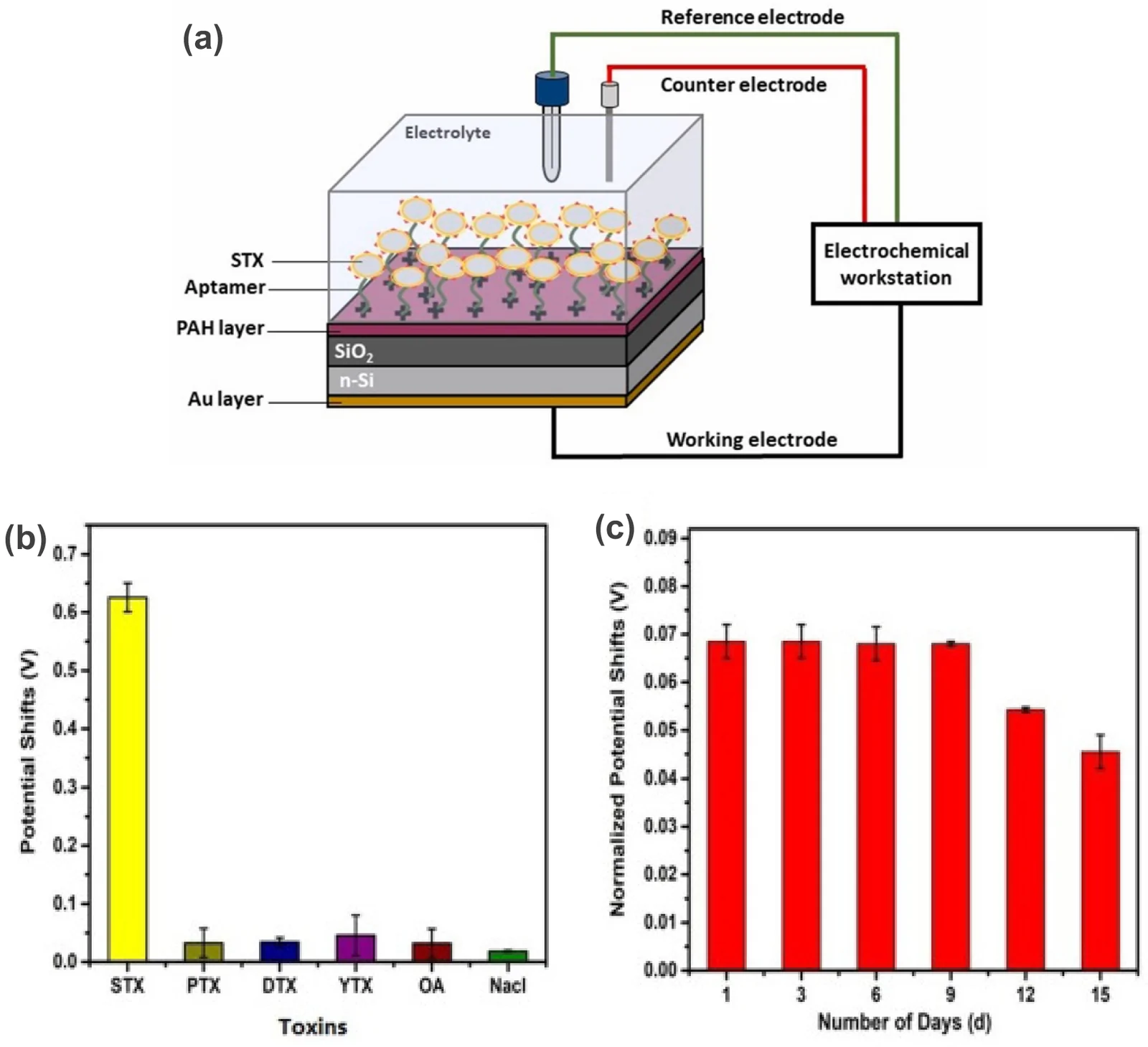
**a** Schematic representation of the ELIS sensor structure and measurement setup. **b** Selectivity analysis of the aptasensor to STX (10 nmol/L), measurement solution (10 nmol/L), PTX (100 nmol/L), DTX-1 (100 nmol/L), YTX (100 nmol/L), and OA (100 nmol/L). **c** Stability analysis of the aptasensor to 10 nmol/L STX on different days. The mean and standard deviation of the 3 experiments are shown. (Figure adapted with permission from Noureen et al., 2022. © 2022 Elsevier)

Noureen et al. developed an ELIS biosensor for the label-free detection of STX, employing anti-STX aptamers electrostatically immobilized on a poly(allylamine hydrochloride) (PAH) layer (Noureen et al., 2022). Figure 4 a presents the schematic of the biosensor and its measurement setup. The biosensor exhibited high specificity for STX and showed a linear response between 0.5 and 100 nmol/L (0.15–30 μg/L) and a LOD of 0.05 nmol/L (0.015 μg/L). Selectivity was confirmed against OA, pectenotoxins (PTX), yessotoxins (YTX), and dinophysistoxin-1 (DTX-1) at tenfold higher concentrations, demonstrating robust discrimination (Fig. 4b). The device also retained 95% of its initial signal for 9 days at 4 °C (Fig. 4c). However, signal decay was observed, likely due to degradation and oxidation of the PAH-modified surface during storage, highlighting the need for stabilization strategies to ensure long-term reliability (Noureen et al., 2022).

The various studies analyzed demonstrate significant advances in the sensitivity, selectivity, and applicability of biosensors for STX detection. Each approach—whether involving antibody immobilization on conductive nanomaterials (Bratakou et al., 2017), cell-based systems that exploit electrophysiological responses (Sun et al., 2023), multisensory potentiometric platforms such as electronic tongues (Cruz et al., 2018; Ferreira et al., 2018) or ELIS devices incorporating aptamers (Noureen et al., 2022; Ullah et al., 2021)—offers distinct advantages and limitations. While potentiometric and electronic tongue sensors stand out for their simplicity and potential for field screening, ELIS systems based on two-dimensional materials provide superior analytical performance, achieving detection limits well below regulatory thresholds. Additionally, aptamer-based platforms generally exhibit greater analytical sensitivity and selectivity, while electronic tongue systems offer broader screening capabilities and greater robustness through chemometric analysis (Cruz et al., 2018; Ferreira et al., 2018; Noureen et al., 2022; Ullah et al., 2021). Cell-based biosensors offer the added advantage of evaluating the electrophysiological effects of sodium channel blockers, although their reliance on living cells increases operational complexity and limits long-term stability (Sun et al., 2023; Vogiazi et al., 2019).

These findings reveal an inherent trade-off between analytical performance and operational simplicity. Selective recognition elements, such as antibodies and aptamers, generally provide greater sensitivity but can suffer degradation and require more elaborate immobilization procedures. In contrast, electronic tongue systems employing synthetic membranes offer greater robustness and lower manufacturing costs, although they are more susceptible to matrix effects and often require multivariate calibration models (Cruz et al., 2018).

However, challenges related to the stability of recognition elements, matrix effects, and the need for recalibration in real-world sample analysis still exist. Most studies have relied on model solutions or artificially contaminated samples, with limited validation on naturally contaminated matrices (Bratakou et al., 2017; Noureen et al., 2022; Sun et al., 2023). Therefore, the integration of advanced materials, more robust immobilization strategies, and predictive calibration models emerges as a promising path to establish these platforms as reliable and sustainable alternatives to conventional STX detection methods. To facilitate comparison between studies and highlight the advantages and disadvantages of different potentiometric approaches, Table 1 summarizes the main recognition strategies, their strengths and limitations, validation with real samples, suitability for field deployment, and estimated readiness for commercialization.

**Table 1:** Comparative characteristics of potentiometric biosensors developed for STX detection

Recognition strategy	Main advantages	Main limitations	Real sample validation	Suitability for field deployment	Commercial readiness
Antibody-based biosensors^1^	High affinity and selectivity	Limited stability and relatively high cost	Yes	Moderate	Medium
Cardiomyocyte-based biosensors^4^	Functional response and toxin discrimination	Complex maintenance and short lifetime	No	Low	Low
Electronic tongue systems^2−3^	Robustness and broad screening capability	Lower specificity and matrix effects	Yes	High	Medium
Aptamer-based biosensors^5^	Excellent sensitivity and selectivity	Complex immobilization procedures and limited long-term stability	Yes	Moderate	Medium

Representative studies: ^1^Bratakou et al.; ^2^Ferreira et al.; ^3^Cruz et al.; ^4^Sun et al.; ^5^Ullah et al., and Noureen et al. Suitability for field deployment and commercialization readiness were critically assessed by the authors based on analytical performance, fabrication complexity, portability, stability, validation using real samples, and scalability considerations

### Impedimetric biosensors

Impedimetric sensors and biosensors operate based on electrochemical impedance spectroscopy (EIS). This technique applies a low-amplitude alternating current (AC) potential across a range of frequencies to the working electrode (Lisdat & Schäfer, 2008). The response provides data on the impedance (Z) and capacitance (C) of the system, which are influenced by the electrochemical properties of the analyte-electrode interface. For a comprehensive analysis of the electrical behavior, equivalent circuit modeling is employed using specialized software. These models simulate the electrochemical processes, allowing for the extraction of key parameters such as charge-transfer resistance (Rct), double-layer capacitance, and Warburg impedance (Lisdat & Schäfer, 2008).

**Fig. 5:**
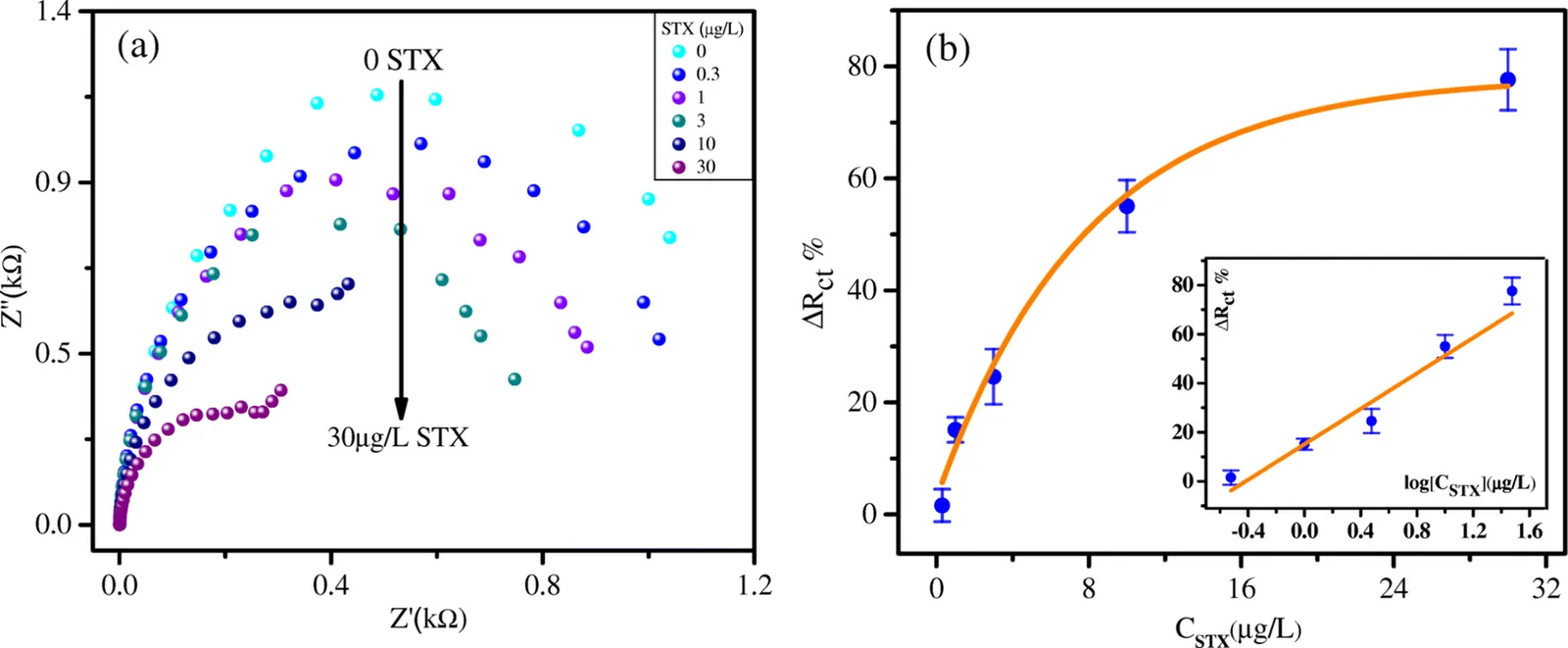
**a** Normalized Nyquist plot of the impedimetric aptasensor after incubation with different STX concentrations: 0.3, 1, 3, 10, and 30 μg/L. **b** Calibration curve for STX, showing the impedance variation rate (∆Rct = 1 − R/R_0_) as a function of STX concentration (CSTX). Inset: linear relationship between ∆Rct and the logarithm of STX concentration [CSTX] (reprinted with permission from Serrano et al., 2021. Copyright 2021 Springer Nature)

Serrano et al. developed an impedimetric biosensor for STX detection, comprising a gold working electrode modified with an aptamer, an Ag/AgCl reference electrode, and a platinum counter electrode (Serrano et al., 2021). EIS measurements were performed in PBS containing 5 mmol/L [Fe(CN)6]^3−/4−^ as the redox probe. The device exhibited a concentration-dependent decrease in Rct within 0.3–30 μg/L (Fig. 5a), attributed to aptamer conformational changes and electrostatic repulsion between STX and the probe. The calibration curve (Fig. 5b) showed a logarithmic relation between *ΔR*_*ct*_ and STX concentration, achieving a LOD of 0.3 μg/L (tenfold below the WHO drinking water guideline). Selectivity tests with microcystin-LR confirmed negligible cross-reactivity. Although the biosensor was validated only in controlled matrices, the study underscored its potential for real-water monitoring, with prospective applications in Peri Lagoon (Brazil), a site recurrently affected by cyanobacterial blooms (Serrano et al., 2021).

Similar to the work of Xiaoting Sun et al. (Sun et al., 2023), Qin Wang et al. developed a cardiomyocyte-based biosensor for the detection of STX and TTX, employing impedance as the transduction principle (Wang et al., 2015). In this platform, cardiomyocyte growth and spontaneous beating activity were monitored in real time, enabling the assessment of cellular responses to neurotoxic exposure. Three parameters were evaluated: cell index, beating rate, and amplitude, each reflecting the physiological state of the cells and their reaction to toxins. The biosensor exhibited remarkable sensitivity, with LOD of 0.087 ng/mL for STX and 89 ng/mL for TTX, based on inhibition of the beating rate. However, as with Sun et al., the study was restricted to controlled conditions and lacked validation with real or complex samples, which remains a critical barrier for translation to environmental or clinical monitoring.

**Fig. 6:**
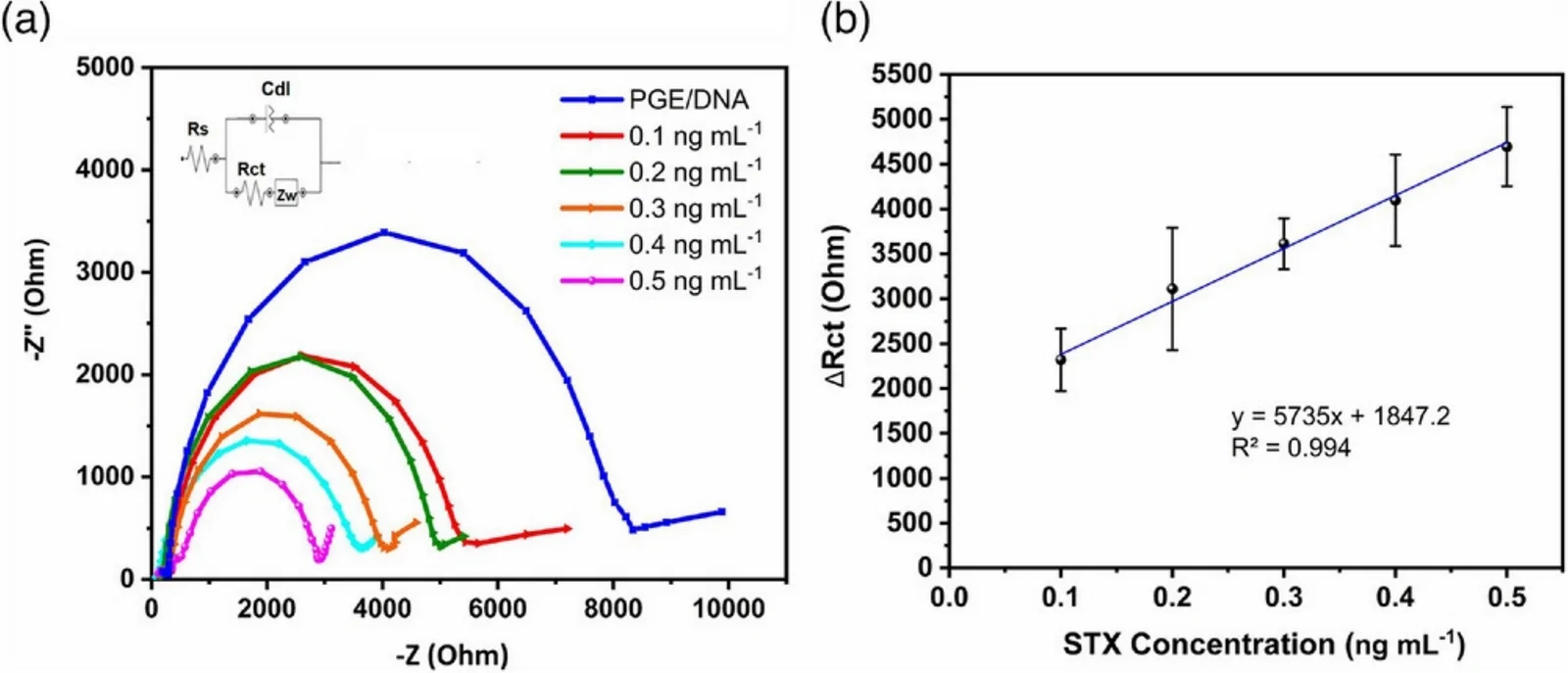
**a** Nyquist diagrams, **b** calibration graph based on EIS results of PGE/DNA/STX in 2.5 mM Fe(CN)_6_^3−/4−^ and 0.1 M KCl aqueous solution with various STX concentrations (0.1–0.5 ng mL^−1^) (reprinted with permission from Kesici-Meco et al., 2025. Copyright 2025 Wiley-VH)

Recently, Kesici-Meco et al. reported the use of EIS for the ultrasensitive detection of STX and cylindrospermopsin (CYN) through the immobilization of double-stranded DNA (dsDNA) on a pencil graphite electrode (PGE) (Kesici-Meco et al., 2025). As illustrated in Fig. 6, Nyquist plots and calibration curves revealed a systematic decrease in *Rct* with increasing STX concentrations. The biosensor achieved detection limits of 0.043 ng/mL for STX and 0.12 ng/mL for CYN, values well below WHO safety thresholds. Notably, the system also demonstrated reliable performance in controlled lake water samples, with a 77% reduction in charge-transfer resistance when exposed to mixed toxins (Kesici-Meco et al., 2025). While these results underscore the promise of PGE/DNA platforms for STX detection, the reliance on controlled matrices still highlights the need for further validation in real, unprocessed water samples. Moreover, unlike aptamers or antibodies, DNA lacks inherent specificity toward STX and CYN, which may limit selectivity under complex field conditions.

The progress of impedimetric platforms for STX detection highlights excellent analytical performance and potential for real-time monitoring. The combination of conductive materials, biorecognition elements, and EIS analysis has enabled detection limits comparable to or even lower than those recommended by regulatory agencies (Kesici-Meco et al., 2025; Serrano et al., 2021; Wang et al., 2015). Aptamer-based platforms have demonstrated excellent selectivity and analytical sensitivity, while cell-based systems offer the added advantage of evaluating the functional effects of sodium channel blockers. DNA-based architectures, in turn, offer simpler and more economical designs, although they may exhibit lower specificity under complex conditions (Kesici-Meco et al., 2025).

However, most devices remain limited to controlled conditions or artificially enriched matrices, restricting their direct applicability to complex environmental samples. Different recognition strategies present distinct trade-offs between sensitivity, stability, and operational complexity. While aptamer-based biosensors generally exhibit superior analytical performance, their long-term stability and surface immobilization still represent challenges. Similarly, cell-based platforms require laborious maintenance and controlled culture conditions, which restricts their practical implementation (Lisdat & Schäfer, 2008; Vogiazi et al., 2019).

In addition, the reliance on degradation-prone biologically derived recognition elements (such as aptamers and living cells) and the lack of standardized field calibration protocols still represent significant challenges. Additional factors, including non-specific adsorption, variations in ionic strength, and limited interlaboratory reproducibility, can also compromise sensor performance in real-world arrays (Lisdat & Schäfer, 2008; Vogiazi et al., 2019). Therefore, the reviewed studies highlight the need to integrate these platforms with validation strategies on real samples and to explore new hybrid materials and more stable bioreceptors, aiming to consolidate impedimetric biosensors as reliable tools for environmental monitoring of STX (Kesici-Meco et al., 2025; Serrano et al., 2021; Wang et al., 2015). To facilitate comparison between studies and highlight the advantages and disadvantages of different impedimetric approaches, Table 2 summarizes the main recognition strategies, their strengths and limitations, validation with real samples, suitability for field application, and predicted commercial readiness.

**Table 2:** Comparative characteristics of impedimetric biosensors developed for STX detection

Recognition strategy	Main advantages	Main limitations	Real sample validation	Suitability for field deployment	Commercial readiness
Aptamer-based biosensors^1^	High sensitivity and selectivity	Surface degradation and immobilization complexity	No	Moderate	Medium
Cardiomyocyte-based biosensors^2^	Functional response and toxin discrimination	Complex maintenance and short lifetime	No	Low	Low
dsDNA-based biosensors^3^	Simple architecture and low cost	Lower specificity under complex matrices	Yes	Moderate	Medium
Label-free impedance sensors^1^	Label-free biorecognition and simple EIS readout	Susceptibility to nonspecific adsorption	No	Moderate	Medium

Representative studies: ^1^Serrano et al. (aptamer-based label-free impedance biosensor); ^2^Wang et al.; ^3^Kesici-Meco et al. Suitability for field deployment and commercialization readiness were critically assessed by the authors based on analytical performance, fabrication complexity, stability, portability, and validation in real samples

### Voltammetric biosensors

Voltammetric sensors and biosensors operate by applying a controlled potential (linear, cyclic, or pulsed) to the working electrode while measuring the corresponding faradaic current (Mahajan et al., 2025). This electrochemical technique allows the quantitative analysis of redox-active species through characteristic oxidation and reduction peaks in the voltammograms. In biosensing, the functionalization of electrodes with recognition elements allows for the selective detection of target analytes, most often through the modulation of redox probe signals. The analytical signal is generally derived from changes in the electron-transfer kinetics of these probes, induced by specific binding events at the electrode interface (Mahajan et al., 2025).

Liu et al. developed a highly sensitive voltammetric biosensor for STX detection by combining in silico peptide screening with electrochemical validation via EIS to identify a high-affinity recognition element (Liu et al., 2024). The selected peptide was then immobilized on a gold electrode modified with reduced graphene oxide (rGO) and gold nanoparticles (AuNPs), which improved electron-transfer efficiency. Under optimized conditions, the biosensor exhibited excellent performance, with a linear detection range of 10–1000 ng/L and a remarkably low LOD of 0.00069 µg/L using differential pulse voltammetry (DPV). Importantly, the biosensor was evaluated not only in seawater and artificial seawater but also in oyster powder samples, achieving recovery rates between 87.3 and 116.2%. These results demonstrate the promise of peptide-based recognition elements as an alternative to traditional antibodies and aptamers (Liu et al., 2024). Nonetheless, despite the encouraging validation in selected food and environmental samples, broader testing across diverse and unprocessed matrices is necessary to confirm robustness and ensure applicability under real-world monitoring scenarios.

More recently, Sequalini et al. (2026) reported a flexible electrochemical biosensor based on photolithographically patterned gold electrodes and anti-STX antibodies immobilized through a simple sodium citrate-assisted strategy (Bernardes Sequalini et al., 2026). Cyclic voltammetry measurements enabled STX detection with a limit of detection of 0.2 μg/L, well below the WHO guideline value for drinking water. In addition, the biosensor exhibited excellent specificity against microcystin-LR and cylindrospermopsin and maintained its analytical performance in mineral water samples with different physicochemical characteristics, demonstrating its potential for environmental monitoring applications (Bernardes Sequalini et al., 2026).

A sensitive and selective voltammetric biosensor for STX detection was reported by Hou et al., based on a gold electrode modified with an octadecanethiol self-assembled monolayer (SAM) and multi-walled carbon nanotubes (MWCNTs) (Hou et al., 2016). STX-specific aptamers were immobilized on the MWCNTs, with methylene blue (MB) acting as the redox probe. Using DPV, the aptasensor achieved a linear detection range of 0.9–30 nM (0.27–9.0 µg/L) and a LOD of 0.38 nmol/L (0.11 µg/L), exceeding regulatory requirements for water safety. High specificity was demonstrated, with no cross-reactivity toward structurally related toxins. Moreover, fortified mussel extract assays yielded recoveries ranging from 63 to 121%, while stability tests showed 90% signal retention after 2 weeks of storage at 4 °C (Hou et al., 2016). However, validation was limited to fortified samples, and further assessment in naturally contaminated shellfish and complex environmental waters remains essential to demonstrate reliability under complex conditions.

**Fig. 7:**
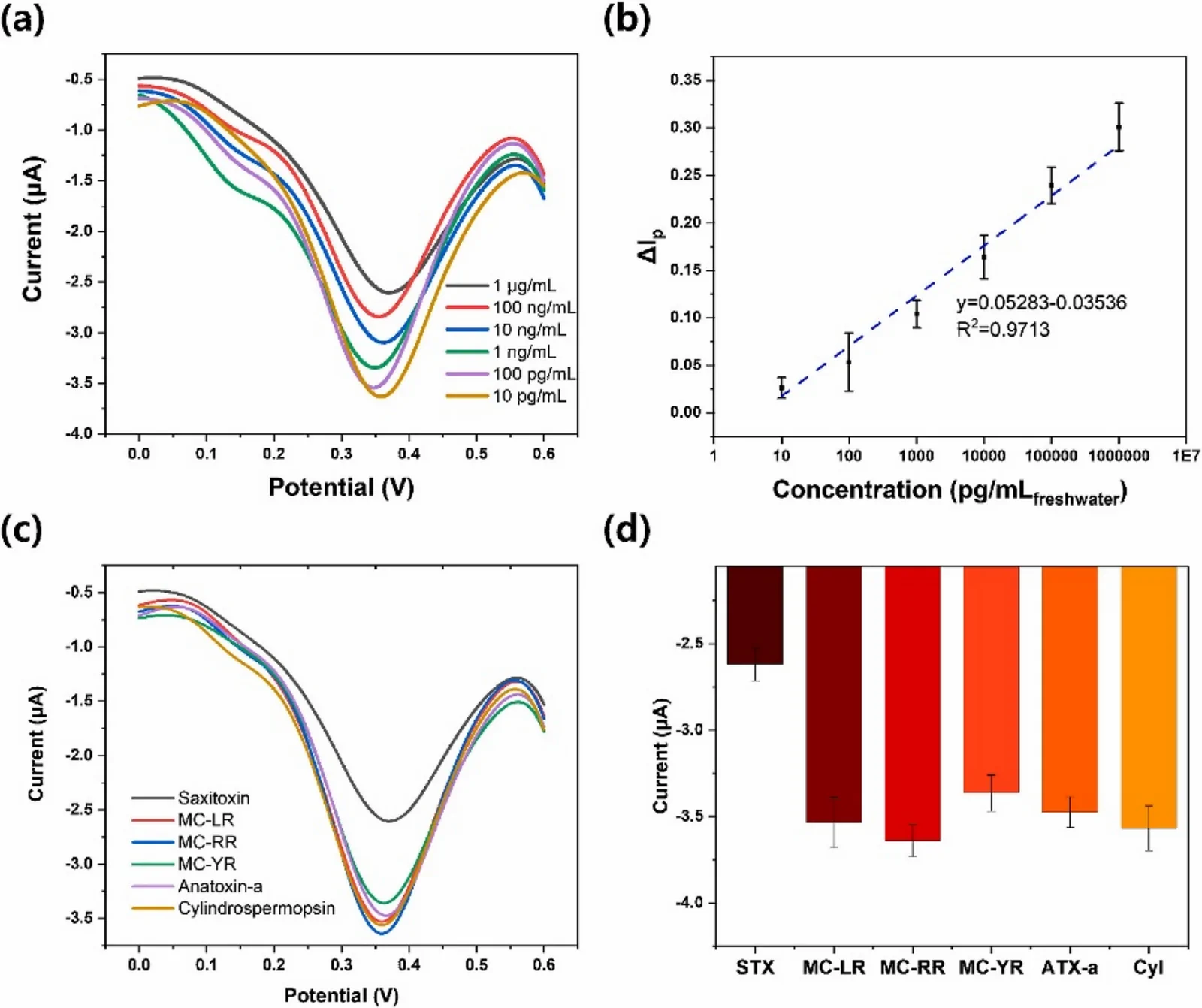
**a** SWV graph of in different concentrations of saxitoxin (in freshwater, 10 pg/mL to μg/mL), **b** linear regression curve of in different concentrations saxitoxin (in freshwater); linear range from 10 pg/mL to 1 μg/mL, **c** SWV graph by toxin for selectivity testing, **d** Peak current at SWV graph based on selectivity (in freshwater) (reprinted with permission from Park et al., 2022, 2022. Copyright 2024 Elsevier)

By integrating a novel round-type micro-gap electrode (RMGE), aptamer recognition, and porous platinum nanoparticles (pPtNPs) for signal amplification, Park et al. developed a voltammetric biosensor for STX detection in freshwater (Park et al., 2022). Using square wave voltammetry (SWV), the sensor achieved outstanding performance, with a LOD of 4.669 pg/mL and a broad linear range (10 pg/mL—10 μg/mL). Figure 7 shows the voltammograms obtained for different STX concentrations, the corresponding calibration curve, and the selectivity test voltammograms with their respective peak currents in freshwater spiked with STX. The biosensor demonstrated excellent selectivity, with negligible interference from other cyanotoxins. However, stability tests were limited, with supplementary data indicating signal retention only up to 4 days, followed by a sharp decline to ~ 40% at day 10. While these results highlight the promise of micro-gap electrode architectures for ultrasensitive detection, the short shelf-life remains a critical barrier for practical application (Park et al., 2022).

Other aptamer-based biosensors for STX detection have also been reported, each demonstrating high analytical performance with distinct nanomaterial modification strategies. Zeng et al. combined a DNA aptamer with silver nanoparticles (AgNPs) and potassium ferricyanide, achieving an LOD of 1 nmol/L (~ 0.3 µg/L) and reliable recoveries (71.2–93.8%) in spiked clam and mantis shrimp samples, with good stability after one week of storage (Zeng et al., 2024). Zheng et al. developed a simple voltammetric aptasensor using methylene blue (MB)-labeled aptamers immobilized on gold electrodes. The device provided a broad detection range from 1 nmol/L to 1 μmol/L and a LOD of 1 nmol/L, with good selectivity against neo-STX, okadaic acid, and common metal ions. However, unlike other aptamer-based platforms, validation in real or complex matrices was not demonstrated, which limits direct assessment of its practical applicability under environmental or food-monitoring conditions (Zheng et al., 2022). Li et al. also employed MB-labeled aptamers immobilized on ordered gold nanopillar arrays, exploiting the high surface area to achieve ultrasensitive detection with a LOD of 1 pM (~ 0.0003 µg/L), nearly four orders of magnitude below regulatory limits, and consistent recovery in shellfish samples (85–112%) (Li et al., 2024). These studies underscore the potential of aptamer-based voltammetric biosensors for STX detection, demonstrating progressive advances in sensitivity and applicability to close to real conditions. However, recurring challenges, most notably matrix interferences and insufficient long-term stability assessments, remain significant barriers to their translation into routine monitoring.

**Fig. 8:**
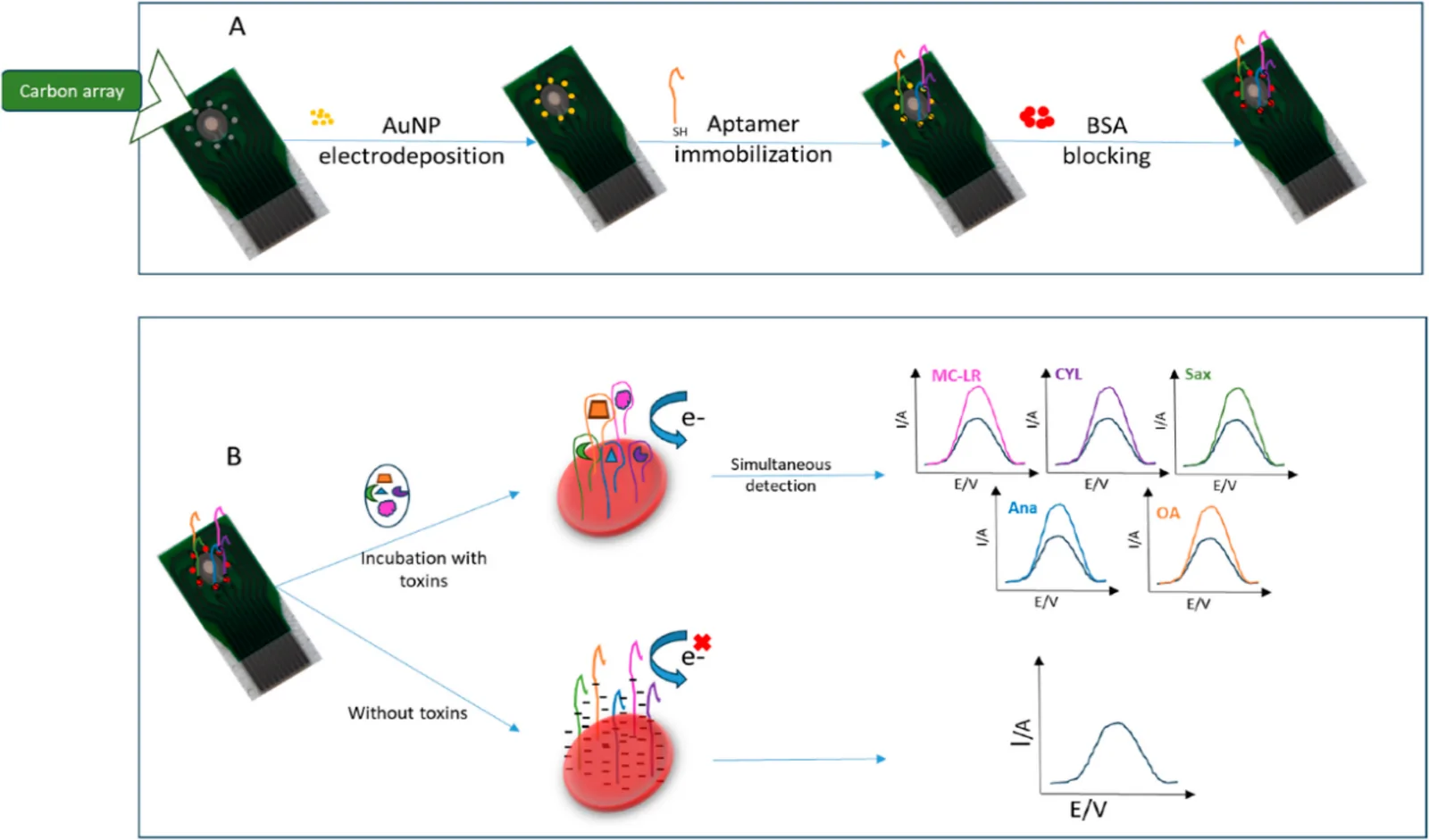
Schematic illustration of the fabrication steps (**A**) and the working principle (**B**) of the multiplexed cyanotoxin aptasensor (reprinted from Rhouati & Zourob, 2024, under the terms of the Creative Commons Attribution License (CC BY 4.0). Copyright 2024 MDPI)

Advances in aptamer-based voltammetric biosensors for STX were further extended by Rhouati and Zourob (2024), who developed a multiplex electrochemical aptasensor capable of simultaneously detecting five cyanotoxins, namely microcystin-LR (MC-LR), CYN, anatoxin-α, STX, and OA (Rhouati & Zourob, 2024). The platform employed an eight-electrode carbon microarray modified with electrodeposited AuNPs, onto which thiolated aptamers specific to each toxin were immobilized. Detection was performed by SWV, monitoring changes in electron transfer induced by aptamer–toxin interactions. The biosensor exhibited ultralow limits of detection for all analytes, including 0.0053 nM for STX, while selectivity studies revealed less than 5% cross-reactivity among non-target toxins (Rhouati & Zourob, 2024). In addition, the device demonstrated excellent reproducibility (RSD < 5%) and maintained stability for up to two months at 4 °C. Validation using laboratory-prepared samples resulted in recoveries ranging from 90 to 109%, highlighting the robustness of the sensing strategy against matrix effects. Compared with single-analyte biosensors, this multiplexed platform represents a significant advance toward comprehensive cyanotoxin monitoring, providing a rapid and cost-effective approach for simultaneous detection. Future validation using naturally contaminated samples will be essential to confirm the applicability of this multiplexed strategy under realistic monitoring conditions (Rhouati & Zourob, 2024) (Fig. 8).

Voltammetric biosensor studies demonstrate the rapid advancement and growing sophistication of platforms for STX detection, driven by the integration of conductive nanomaterials and molecular recognition elements such as aptamers and peptides, which significantly enhance signal amplification and selectivity (Hou et al., 2016; Liu et al., 2024; Park et al., 2022). The incorporation of microelectrodes, metallic nanoparticles, and multiplexed array architectures has enabled ultralow detection limits, often several orders of magnitude below regulatory thresholds, and even simultaneous analysis of multiple cyanotoxins (Zeng et al., 2024; Li et al., 2024; Rhouati & Zourob, 2024; Zheng et al., 2022). For instance, peptide- and aptamer-based configurations have achieved exceptional sensitivities and reliable recoveries in food and water samples (Hou et al., 2016; Liu et al., 2024), while multiplexed microarray systems have allowed the concurrent detection of up to five toxins with high reproducibility and minimal cross-reactivity (Rhouati & Zourob, 2024).

A comparison among the reported voltammetric platforms reveals that aptamer-based biosensors dominate the field owing to their excellent sensitivity, high selectivity, and compatibility with diverse signal amplification strategies. Peptide-based sensors represent a promising alternative, offering simpler synthesis and potentially improved stability while maintaining satisfactory analytical performance (Liu et al., 2024). More recently, antibody-based flexible platforms have emerged as attractive candidates for practical applications owing to their straightforward fabrication procedures and favorable balance between analytical performance and manufacturing simplicity (Bernardes Sequalini et al., 2026). In addition, nanomaterials such as AuNPs, AgNPs, rGO, MWCNTs, and ordered nanopillar arrays play a fundamental role in enhancing electron-transfer kinetics and increasing the effective surface area available for bioreceptor immobilization, thereby contributing to the remarkable analytical sensitivity achieved by these devices (Hou et al., 2016; Li et al., 2024; Zheng et al., 2022).

Despite these promising results, most devices have been validated only in spiked samples rather than naturally contaminated matrices, limiting their immediate applicability to real environmental conditions (Park et al., 2022; Zeng et al., 2024; Li et al., 2024). Furthermore, significant challenges remain regarding long-term stability, batch-to-batch reproducibility, fabrication complexity, and manufacturing costs (Rhouati & Zourob, 2024; Vogiazi et al., 2019). Platforms still require sample pretreatment procedures and controlled experimental conditions, which restrict their implementation in continuous or on-site monitoring applications (Hou et al., 2016; Park et al., 2022; Zeng et al., 2024)**.** Therefore, although voltammetric biosensors currently represent the most mature and analytically sensitive electrochemical approach for STX detection, their successful translation into practical analytical tools will depend on further progress in standardization, robustness, and validation under field-relevant conditions (Rhouati & Zourob, 2024; Vogiazi et al., 2019). To facilitate cross-study comparison and highlight the trade-offs among different voltammetric approaches, Table 3 summarizes the principal recognition strategies, strengths and limitations, validation using real samples, suitability for field deployment, and estimated commercialization readiness.

**Table 3:** Comparative characteristics of voltammetric biosensors developed for STX detection

Recognition strategy	Main advantages	Main limitations	Real sample validation	Suitability for field deployment	Commercial readiness
Aptamer-based biosensors^1−5^	Excellent sensitivity and selectivity	Surface degradation, matrix effects, and multistep functionalization	Yes	Moderate	Medium
Peptide-based biosensors^6^	Simple synthesis and satisfactory stability	Limited number of validated receptors	Yes	Moderate	Medium
Antibody-based biosensors^7^	High specificity and straightforward immobilization procedures	Dependence on antibody stability and relatively long incubation times	Yes	High	Medium
Multiplexed aptasensors^8^	Simultaneous detection of multiple toxins with high reproducibility	Fabrication complexity and higher cost	Yes	High	Medium
Micro-gap electrode platforms^4^	Exceptional sensitivity and low sample consumption	Short shelf-life and limited long-term stability	Yes	Moderate	Low

Representative studies: ^1^Hou et al.; ^2^Zeng et al.; ^3^Li et al.; ^4^Park et al.; ^5^Zheng et al.; ^6^Liu et al.; ^7^Sequalini et al.; ^8^Rhouati and Zourob. Suitability for field deployment and commercialization readiness were critically assessed by the authors based on analytical performance, fabrication complexity, portability, stability, validation using real samples, and scalability considerations

**Fig. 9:**
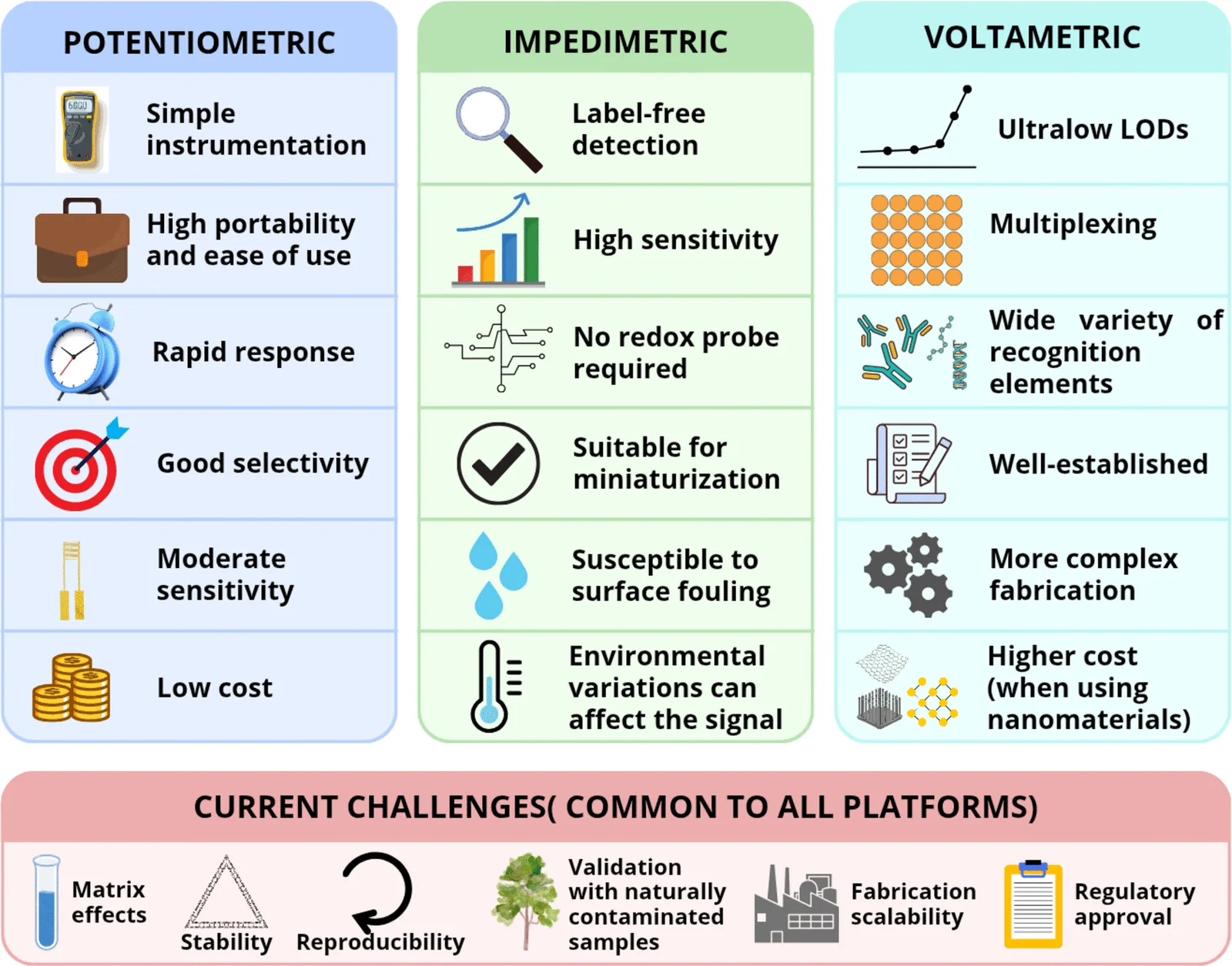
Comparative overview of potentiometric, impedimetric, and voltammetric biosensors for STX detection, highlighting their principal characteristics, strengths, limitations, and common challenges affecting their practical implementation and commercialization

A comparative analysis of the three electrochemical transduction strategies reveals distinct strengths and limitations associated with each approach. As illustrated in Fig. 9, potentiometric biosensors are characterized by their simplicity and portability, impedimetric platforms provide label-free operation and excellent analytical performance, whereas voltammetric sensors offer ultralow detection limits and multiplexing capabilities. Nevertheless, matrix effects, reproducibility, validation using naturally contaminated samples, fabrication scalability, and regulatory approval remain common challenges that continue to hinder the practical implementation and commercialization of these technologies.

## Main challenges for STX detection

Sample preparation remains a major bottleneck in STX analysis across conventional and emerging platforms. In freshwater systems, most toxins remain intracellular during bloom stages, requiring prior cell lysis (e.g., freeze–thaw cycles, sonication, or chemical disruption), followed by solvent extraction and, frequently, cleanup steps such as solid-phase extraction to remove pigments and organic matter (Wiese et al., 2010). Similarly, in seafood matrices, STX accumulates in bivalve tissues, demanding homogenization and multi-step solvent extraction prior to chromatographic or immunochemical analysis (Turner et al., 2020). Although essential for accurate toxin recovery, these procedures significantly increase analysis time and cost, while introducing variability related to extraction efficiency and matrix effects (Wiese et al., 2010). These limitations highlight the need for alternative analytical strategies, such as electrochemical biosensors, which could potentially reduce or eliminate extensive sample preparation and enable faster, more accessible STX detection (Ronkainen et al., 2010).

The main challenges in the electrochemical biosensing of STX revolve around four critical aspects: stability, selectivity, validation, and robustness.

The stability of biorecognition elements such as aptamers, antibodies, and cells still limits the lifetime and reliability of biosensors, particularly under non-controlled conditions (Hou et al., 2016; Serrano et al., 2021). Selectivity also remains a major issue since STX structural analogs and interfering compounds are often present in complex environmental and food matrices (Bratakou et al., 2017; Cruz et al., 2018). Most reported studies rely on spiked samples, with few validations carried out using naturally contaminated matrices, which restricts the practical applicability of the sensors (Kesici-Meco et al., 2025; Liu et al., 2024; Noureen et al., 2022). Finally, robustness under real-world conditions is often compromised by the presence of salts, proteins, and pigments that can alter the electrochemical response (Park et al., 2022; Rhouati & Zourob, 2024). These challenges highlight the need for strategies aimed at improving the stability and selectivity of bioreceptors, as well as comprehensive validation in complex environmental and biological matrices.

Another important aspect concerns the role of nanomaterials in the analytical performance of electrochemical STX biosensors. Materials such as AuNPs, AgNPs, rGO, MWCNTs, and ordered nanopillar arrays have been employed to increase the active surface area, facilitate electron transfer, and improve the immobilization efficiency of biorecognition elements (Hou et al., 2016; Li et al., 2024; Liu et al., 2024; Zeng et al., 2024). Although these nanostructured interfaces have enabled low detection limits and remarkable analytical sensitivity, challenges associated with synthesis reproducibility, batch-to-batch variability, and long-term stability still hinder their large-scale implementation and commercialization.

Beyond analytical performance, practical aspects associated with the transition from laboratory prototypes to commercial devices remain insufficiently explored. Issues related to scalable electrode fabrication, quality control, storage requirements, calibration standardization, and interlaboratory reproducibility still represent significant barriers to the widespread adoption of electrochemical STX biosensors. In addition, regulatory approval requires extensive validation using naturally contaminated matrices and comparison with established methods such as LC–MS/MS and ELISA, which are currently considered reference techniques for PSTs analysis (Turner et al., 2020; Vogiazi et al., 2019). Recent studies have demonstrated that simplified immobilization procedures and flexible electrode architectures may contribute to overcoming some of these barriers. For example, flexible gold-based biosensors fabricated by photolithographic methods and functionalized through single-step immobilization strategies have shown promising analytical performance while maintaining compatibility with scalable manufacturing processes (Bernardes Sequalini et al., 2026). Therefore, future developments should focus on enhancing robustness, reproducibility, manufacturing feasibility, and regulatory compliance.

**Fig. 10:**
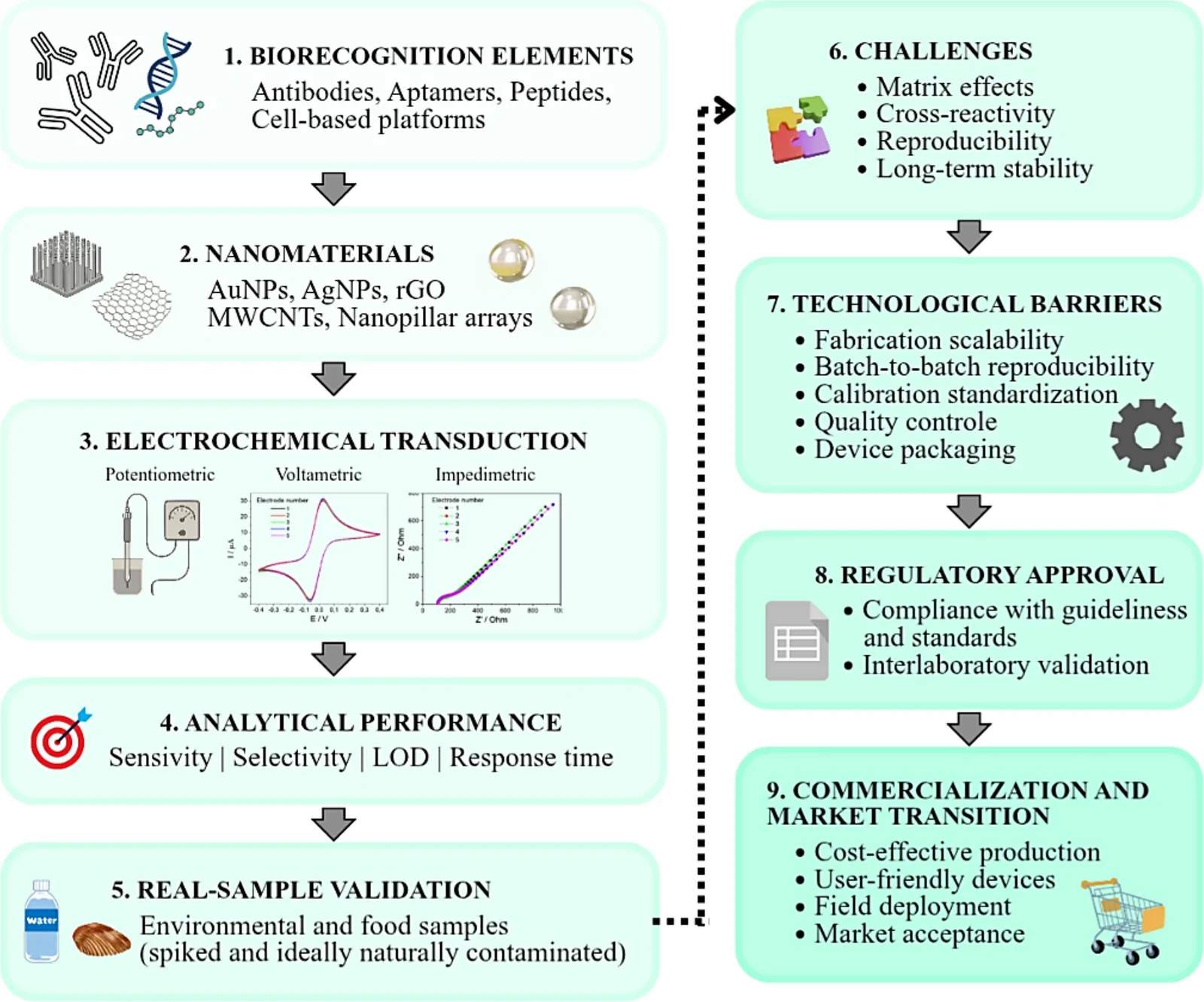
Roadmap illustrating the progression of electrochemical STX biosensors from biorecognition and nanomaterial engineering to analytical performance, real-sample validation, technological barriers, regulatory approval, and eventual commercialization and market transition

Figure 10 summarizes the progression of electrochemical STX biosensors from biorecognition and nanomaterial engineering to analytical performance, real-sample validation, technological barriers, regulatory approval, and eventual commercialization. The scheme highlights that market transition requires not only excellent analytical characteristics but also improvements in reproducibility, standardization, scalability, and regulatory compliance.

Several analytical and practical limitations still hinder the reliable detection and widespread implementation of electrochemical STX biosensors, including matrix effects, instability of biorecognition layers, limited reproducibility, and the lack of standardized validation protocols. To provide a systematic overview of these aspects, Table 4 summarizes the principal challenges currently affecting the practical implementation and commercialization of STX biosensors, together with their impact on analytical performance and market transition.

**Table 4:** Main challenges associated with electrochemical STX biosensors and their impact on practical implementation and commercialization

Main Challenge	Impact on sensor performance and commercialization
Matrix effects and sample interferences^1,2,3^	Reduced selectivity and analytical accuracy in complex matrices
Cross-reactivity among PSTs analogues^4,5^	Difficulty in discriminating individual toxins
Stability of biorecognition elements^6,7^	Reduced shelf-life and long-term reliability
Reproducibility between batches^1,3^	Limited large-scale manufacturing and poor interlaboratory agreement
Limited validation using naturally contaminated samples^1,2,3^	Reduced confidence in field applications
Fabrication scalability and manufacturing complexity^3^	Increased production cost and difficulty in market transition
Calibration standardization^8^	Poor comparability among studies and devices
Quality control and packaging requirements^8^	Difficulties in product certification and storage
Regulatory approval and interlaboratory validation^9^	Delayed commercialization and implementation
Competition with conventional techniques (LC–MS/MS and ELISA)^8,9^	Slower adoption of biosensor technologies despite their portability and rapid response

Representative studies: ^1^Zeng et al.; ^2^Rhouati and Zourob; ^3^Sequalini et al.; ^4^Hou et al.; ^5^Zheng et al.; ^6^Ullah et al.; ^7^Vogiazi et al.; ^8^Vogiazi et al.; ^9^Turner et al. The impacts summarized in this table were critically assessed by the authors based on the studies reviewed and on current challenges associated with the transition of electrochemical STX biosensors from proof-of-concept devices to commercially viable analytical platforms

The detection of STX remains a critical challenge for environmental monitoring and food safety, as it can lead to paralytic shellfish poisoning, a syndrome characterized by symptoms such as tingling, numbness, muscle weakness, and, in severe cases, respiratory failure and death (Arnich & Thébault, 2018). This review highlights progress in the development of electrochemical biosensors for STX detection, covering potentiometric, impedimetric, and voltammetric platforms. The studies analyzed employed various biorecognition strategies, including peptides, cardiomyocytes, antibodies, and, most notably, aptamers, which were present in the majority of the devices. Research by Bratakou et al. (2017) and Ullah et al. (2021) demonstrates that modifications with nanomaterials, such as graphene, metal nanoparticles, and 2D materials, significantly enhance the analytical performance of the sensors.

Electrochemical biosensors represent a promising alternative to conventional methods, offering high sensitivity, selectivity, portability, and miniaturization potential (Ronkainen et al., 2010; Vogiazi et al., 2019). Despite substantial technological advances, important barriers related to scalability, regulatory approval, and manufacturing reproducibility still limit widespread implementation, comprehensive validation under real environmental and food monitoring conditions remains limited, restricting confidence in routine field deployment.

Given this scenario, there is a clear need for further investigations to overcome current limitations, such as the stability of biorecognition elements and the simplification of manufacturing processes. Future studies should focus on device miniaturization, improving robustness and enabling field applications, particularly for in situ monitoring of contaminated water samples. These advances are essential to meet international guidelines set by the WHO, which establish a provisional limit of 3.0 µg/L for total saxitoxins in drinking water (Chorus & Welker, 2021), ensuring global water safety.

## Conclusions and perspectives

Electrochemical biosensors have emerged as powerful analytical tools for the detection of saxitoxin, offering excellent analytical performance, fast response times, and compatibility with portable and miniaturized platforms. Recent advances demonstrate that detection limits below regulatory thresholds are achievable across potentiometric, impedimetric, and voltammetric transduction strategies, highlighting their potential for environmental and food safety monitoring. Moreover, the incorporation of nanomaterials and diverse biorecognition elements, including antibodies, aptamers, peptides, and cell-based platforms, has expanded the analytical capabilities of these sensing platforms.

Nevertheless, a comparison of the literature reveals substantial variability in device architectures, validation protocols, and testing conditions, thereby limiting direct benchmarking and real-world extrapolation. Most reported studies remain focused on proof-of-concept demonstrations under controlled conditions, underscoring a gap between laboratory performance and operational deployment. Analytical performance alone is insufficient to determine the practical applicability of a given platform, as factors such as stability, reproducibility, fabrication complexity, and validation using naturally contaminated samples are equally important for successful implementation. Although remarkable progress has been achieved, future efforts should prioritize the translation of laboratory-scale developments into robust analytical platforms capable of meeting regulatory, manufacturing, and operational requirements.

Future progress in this field will depend on the consolidation of robust biointerfaces, improved tolerance to complex sample matrices, and harmonized reporting and validation practices. Aligning technological development with analytical validation and regulatory requirements will be essential to translate electrochemical STX biosensors from experimental systems into reliable tools for routine monitoring and early-warning applications. Emerging trends such as multiplexed detection, flexible and miniaturized sensing platforms, simplified immobilization strategies, and integration with portable electronics are expected to further accelerate the development of field-deployable devices. Recent advances indicate that electrochemical STX biosensors are progressively moving beyond proof-of-concept demonstrations toward scalable and commercially relevant sensing technologies.

## Data Availability

No datasets were generated or analyzed during the current study.

## Declarations

All authors have read, understood, and complied, as applicable, with the statement on “Ethical Responsibilities of Authors” found in the Instructions for Authors.
